# Hes1 expression in mature neurons in the adult mouse brain is required for normal behaviors

**DOI:** 10.1038/s41598-019-44698-y

**Published:** 2019-06-03

**Authors:** Tadanobu Matsuzaki, Toru Yoshihara, Toshiyuki Ohtsuka, Ryoichiro Kageyama

**Affiliations:** 1 Institute for Frontier Life and Medical Sciences, Kyoto University, Kyoto, 606-8507 Japan; 2 Graduate School of Medicine, Kyoto University, Kyoto, 606-8501 Japan; 3 Medical Research Support Center, Graduate School of Medicine, Kyoto University, Kyoto, 606-8501 Japan; 4 Graduate School of Biostudies, Kyoto University, Kyoto, 606-8501 Japan; 5 Institute for Integrated Cell-Material Sciences (iCeMS), Kyoto University, Kyoto, 606-8501 Japan

**Keywords:** Fear conditioning, Social behaviour

## Abstract

Hes1 regulates the maintenance and proliferation of neural stem/progenitor cells as an essential effector of the Notch signaling pathway. Although Notch signaling is also involved in the functions of mature neurons in learning and memory and in the risk factors for mental disorders such as schizophrenia and bipolar disorder, the *in-vivo* role of Hes1 in mature neurons remains unknown. Here, we found that Hes1 is expressed by subsets of both excitatory and inhibitory neurons in the adult mouse brain, and that Hes1 expression is induced by neuronal stimulation. Furthermore, inactivation of *Hes1* in excitatory neurons resulted in abnormal fear and anxiety behaviors concomitantly with higher neuronal excitability in the amygdala, while inactivation of *Hes1* in inhibitory neurons resulted in increased sociability and perseverative tendencies. These results indicated that Hes1 is functionally important for normal behaviors not only in excitatory neurons but also in inhibitory neurons in the adult brain.

## Introduction

Notch signaling regulates the maintenance and proliferation of neural stem/progenitor cells and glial versus neuronal differentiation in the developing nervous system^1^. Recent studies suggest that Notch signaling is also involved in neuronal activity during learning and memory^2–5^ and in mental disorders such as schizophrenia and bipolar disorder^6–10^. Indeed, the Notch intracellular domain (NICD), an active form of the Notch protein, is expressed by subsets of neurons in many regions of the brain, including the cerebral cortex and hippocampus, and inactivation of Notch signaling in the postnatal hippocampal neurons leads to memory defects^4,5^. However, the mechanism by which Notch signaling regulates such neuronal activities remains unknown.

Notch is a transmembrane protein, which upon activation by its ligands, is cleaved at the transmembrane region, releasing NICD. NICD is then transferred into the nucleus, where it forms a complex with the DNA-binding factor Rbpj and the transcriptional co-activator Mastermind-like^1^. This complex activates the expression of various downstream genes such as *Hes1* and *Hes5*, which encode basic helix-loop-helix transcriptional repressors^1^. As an essential Notch signaling effector, Hes1 plays important roles in the maintenance and proliferation of neural stem/progenitor cells and glial versus neuronal differentiation in the developing nervous system^11–13^. It was previously shown that Hes1 is also expressed by cultured neurons and controls the neuronal activity^14^. Hes1 represses the expression of GluR1, an AMPA receptor required for synaptic plasticity, and thereby inhibits GluR1-mediated calcium influx in cultured cortical neurons^14^. Furthermore, Hes1 antagonizes the effects of β-amyloid and increases the survival of cultured hippocampal neurons^15^. These results provide evidence that Hes1 regulates *in vitro* neuronal activity, but the *in vivo* functions of Hes1 in the adult brain still remain to be analyzed.

To understand the role of Hes1 in mature neurons in the adult brain, we examined Hes1 expression in the adult mouse brain. Furthermore, we genetically ablated the Hes1 gene in mouse neurons. Because conventional *Hes1* knock-out (KO) leads to premature death^16^, we generated *Hes1* conditional KO (cKO) mice by taking advantage of Cre/LoxP recombination system. We crossed *Hes1* flox mice with either Math2-Cre mice or Gad2-Cre mice, which express Cre recombinase in excitatory or inhibitory neurons, respectively. *Hes1* is therefore deleted in all excitatory neurons in the former cKO mice, and it is deleted in all inhibitory neurons in the latter cKO mice. We performed behavior analyses of these cKO mice and found that Hes1 is functionally important for normal behaviors not only in excitatory neurons but also in inhibitory neurons in the adult brain.

## Results

### Hes1 is expressed by both excitatory and inhibitory neurons

**Figure 1:**
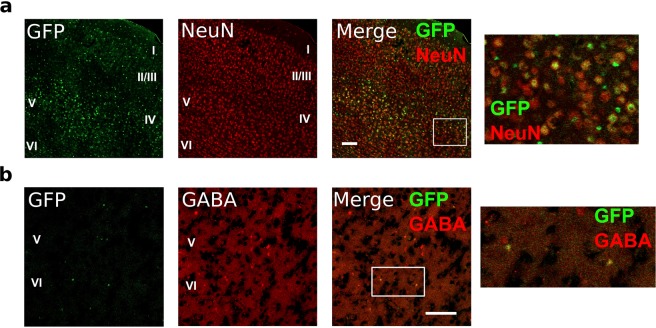
Hes1 expression in neurons in the adult mouse brain (10 weeks old). (**a**) Double immunofluorescence staining of GFP (green, with TSA amplification) and NeuN (red) in a coronal section of the primary sensory cortex of a Venus-Hes1 knock-in mouse. (**b**) Double immunofluorescence staining of GFP (green, without TSA amplification) and GABA (red) in a coronal section of the primary motor cortex of a Venus-Hes1 knock-in mouse. Boxed regions are enlarged on the right. Cortical layers I-VI are labeled. Scale bars = 100 µm.

We first analyzed the expression pattern of Hes1 in the cerebral cortex of adult mice. To this end, we used Venus-Hes1 fusion knock-in mice, in which Venus (GFP variant) cDNA was knocked-in in-frame into the 5′ region of the Hes1 gene so that a Venus-Hes1 fusion protein was expressed^17^. In these mice, Venus expression, detected with a GFP antibody, correlated very well with endogenous Hes1 (ref.^17^). In the cortex of adult mice, Hes1 was expressed at low levels in subsets (approximately 3–15%) of NeuN^+^ neurons (Fig. 1a).

We next tried to perform immunostaining for Venus-Hes1 and excitatory pyramidal neuron-specific markers, but it was technically difficult (data not shown), because Venus-Hes1 expression levels were very low in neurons. To overcome this difficulty, we adopted an alternative approach. *Venus-Hes1* floxed;*Hes1* floxed mice^17,18^ were crossed with Math2-Cre mice, in which Cre is expressed specifically in post-mitotic excitatory neurons from around embryonic day (E)11 (ref.^19^). In the resultant *Venus-Hes1* floxed;*Hes1* floxed;Math2-Cre mice, residual Venus expression occurred under the control of the endogenous *Hes1* promoter in Cre-positive cells (Math2-expressing excitatory neurons) (Supplementary Fig. S1a). Hes1 expression is normally kept at very low levels by negative feedback, in which Hes1 represses its own expression by directly binding to its promoter^20^. However, because there was no Hes1 protein in these Cre-positive cells (Supplementary Fig. S1a), Venus protein should be overexpressed without negative feedback. Indeed, Venus protein was clearly expressed in many neurons in the cortex (Supplementary Fig. S1b) and the hippocampus (Supplementary Fig. S1c). Higher magnification showed that Venus was highly expressed in many pyramidal neurons in the cerebral cortex of adult mice (Supplementary Fig. S1d). Thus, Hes1 is expressed by Math2-expressing excitatory neurons *in vivo*, but is maintained at very low levels by negative feedback. Low levels of Hes1 were also detected in subsets (approximately 30%) of GABA^+^ inhibitory neurons in the cortex (Fig. 1b). These results indicated that Hes1 is expressed by subsets of both excitatory and inhibitory neurons in the adult mouse brain.

### Hes1 expression is induced in neurons by stimulation

**Figure 2:**
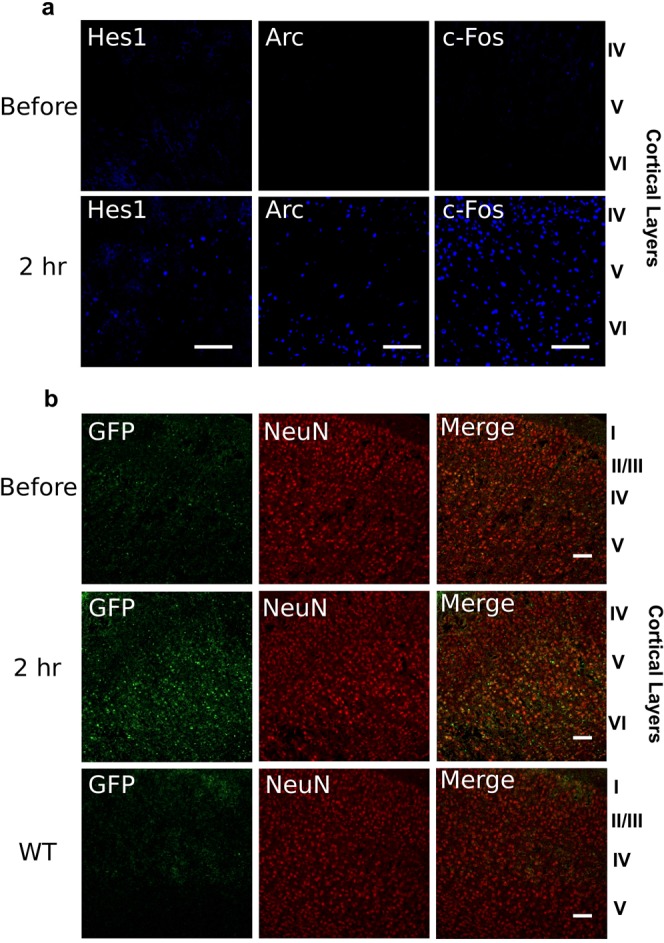
Induction of Hes1 expression by neuronal stimulation. (**a**) Immunofluorescence staining for Hes1 (left panels), Arc (middle panels), and c-Fos (right panels) in the primary visual cortex of wild-type mice before (upper panels) and 2 hours after (lower panels) visual stimulation. (**b**) Double immunofluorescence staining for GFP (left panels), NeuN (middle panels), and merged images (right panels) in the primary sensory cortex of Venus-Hes1 knock-in mice before (upper panels) and 2 hours after (middle panels) electroconvulsive stimulation. Wild-type (WT) mice were shown as a negative control of immunostaining for GFP (lower panels). Cortical layers I-VI are labeled on the right. Scale bars = 100 μm.

The Hes1 expression patterns in cortical neurons differed among mice, which led us to suspect that Hes1 expression is temporally dynamic and induced by certain stimuli. It is well known that immediate early genes (IEG), such as Arc and c-Fos, are expressed promptly after cell stimulation^21^. To test the possibility that Hes1 expression might be also induced by neuronal stimulation, we focused on the visual cortex and assessed whether Hes1 expression can be induced by visual stimulation. When mice were kept in the dark for three days, there were only few Hes1^+^ neurons in the visual cortex (Fig. 2a, upper panel). Furthermore, Arc and c-Fos were mostly negative in this region (Fig. 2a, upper panels). However, both Arc and c-Fos expression were robustly induced 2 hours after light stimulation (~70-fold and ~30-fold increase, respectively, in cell number) (Fig. 2a, lower panels), consistent with the previous reports^22,23^. Hes1 expression was also induced at the same time point, although in fewer cells (~5-fold increase in cell number) (Fig. 2a).

We next performed electro-convulsive stimulation, another well-known method to induce IEG expression^24,25^, to see whether broader stimulation of cortical neurons leads to broader expression of Hes1. Trans-cranial electric stimulation was administered to mice, who exhibited general seizures. Two hours after electric stimulation, in addition to Arc and c-Fos expression (data not shown), Hes1 expression was broadly induced in NeuN^+^ neurons in the cerebral cortex (~2-fold increase in cell number) (Fig. 2b). These results indicated that Hes1 expression can be induced in neurons by stimulation.

### Abnormal fear and anxiety in excitatory neuron-specific *Hes1* cKO mice

**Figure 3:**
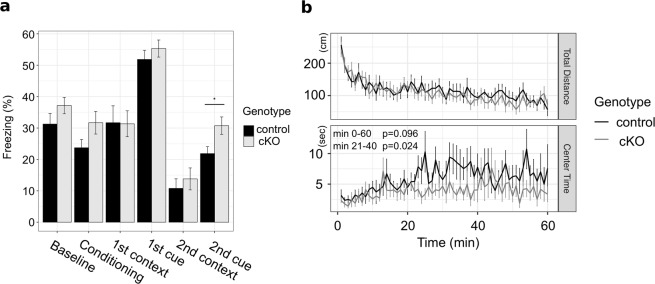
Inactivating *Hes1* in excitatory neurons leads to abnormal retention of fear memory and a slight increase in anxiety level. (**a**) Bar plots showing freezing time of wild-type control (black, n = 17) and Math2-Cre;*Hes1* cKO (gray, n = 17) mice during fear conditioning, context recall, and cued recall of the fear conditioning test as well as before fear conditioning (baseline). Interval between 1st and 2nd tests was 6 months. Data are presented as mean ± SEM; *p < 0.05; two tailed Student’s *t*-test. (**b**) Line plots showing total distance traveled and time spent in the center during the open field test for wild-type control (black, n = 17) and *Hes1* cKO (gray, n = 17) mice. Data are presented as mean ± SEM; the p-values indicate a genotype effect in the two-way repeated measures ANOVA.

To analyze the function of Hes1 in the adult brain, we deleted *Hes1* specifically from excitatory neurons. To this end, we mated the Math2-Cre driver line with the *Hes1*-floxed line, in which loxP sequences are located at the first intron and 3′ UTR of the Hes1 gene (Supplementary Fig. S1a)^18^. The resulting cKO (Math2-Cre;*Hes1* cKO) mice (10 weeks old) were then subjected to a series of behavioral analyses. These cKO mice displayed no significant difference in general health, spatial memory, working memory, sociability, depression-like behavior, or sensorimotor gating, compared to wild-type control mice (Supplementary Figs S2–S4). Furthermore, the freezing response at the baseline level was not significantly different between the wild type and cKO mice (Fig. 3a, baseline). However, we found that the cKO mice displayed abnormal behaviors in the following tests.

In the fear conditioning test, these cKO mice were moved to a chamber and conditioned to associate an auditory cue with a foot shock (see Methods). The next day, they were placed in the same chamber without an auditory cue (contextual recall test) or in a different chamber with an auditory cue (cued recall test). Math2-Cre;*Hes1* cKO mice exhibited no significant differences in freezing time in the first contextual recall test or in the cued recall test, compared to wild-type control mice (Fig. 3a). The second contextual recall and cued recall tests were performed six months later. While Math2-Cre;*Hes1* cKO mice still exhibited no significant difference in the second contextual recall test compared with wild-type control mice, they did exhibit significantly longer freezing time in the second cued recall test than did the wild-type control mice (Fig. 3a). These results indicated that the mutant mice exhibited abnormal retention of fear memory in an auditory cued test.

In an open field test, mice were placed in a bright open-field apparatus (see Methods). While the total traveled distance was not significantly different between Math2-Cre;*Hes1* cKO and wild-type control mice (Fig. 3b, upper panel), the cKO mice spent a significantly shorter time in the center area than did the wild-type control mice (Fig. 3b, lower panel), suggesting that they had a mild increase in anxiety levels. Thus, inactivation of *Hes1* in excitatory neurons led to abnormal fear and anxiety, suggesting that Hes1 plays an important role in the function of post-mitotic excitatory neurons in the adult brain.

### Higher excitability in the amygdala of excitatory neuron-specific *Hes1* cKO mice

**Figure 4:**
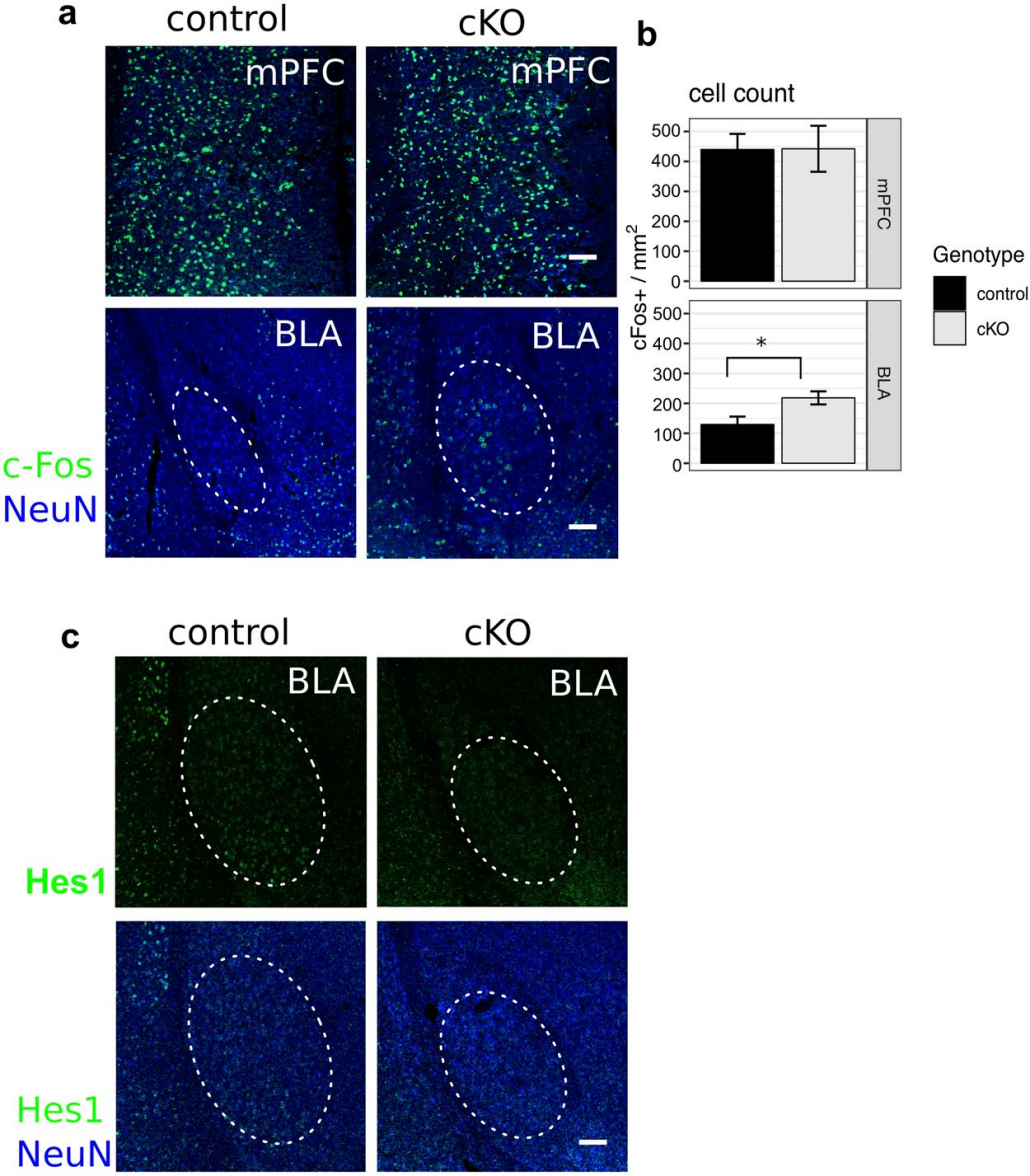
Cell excitability in the amygdala differs in excitatory neuron-specific Hes1 cKO mice. (**a**) Double immunofluorescence staining for c-Fos (green) and NeuN (blue) in the medial pre-frontal cortex (mPFC, upper panels) and basolateral amygdala (BLA, lower panels) of wild-type control and Math2-Cre;*Hes1* cKO mice. We first performed fear conditioning experiment, and then examined c-Fos expression 2 hours after the second cued recall test. Scale bars = 100 µm. (**b**) Bar graphs showing the density of c-Fos-positive neurons in mPFC and BLA of wild-type control (black, n = 4) and Math2-Cre;*Hes1* cKO (gray, n = 4) mice. Data are presented as mean ± SEM; *p < 0.05; two tailed Student’s *t*-test. (**c**) Double immunofluorescence staining for Hes1 (green) and NeuN (blue) in the BLA of wild-type control and Hes1 cKO mice 2 hours after the auditory cue. Scale bar = 100 µm.

Behavioral analyses suggested that Math2-Cre;*Hes1* cKO mice had increased fear retention and anxiety levels. To determine the brain regions responsible for these abnormal fear and anxiety behaviors, we compared the neuronal excitabilities of the cKO and wild-type control mice. In particular, we focused on the medial pre-frontal cortex (mPFC) and amygdala - two regions that are reported to be important for fear memory^26–30^. We examined c-Fos expression to visualize the neuronal excitability in these two regions after the second recall with auditory cue in the fear conditioning test described above. In the mPFC, there was no significant difference in c-Fos expression levels between Math2-Cre;*Hes1* cKO and wild-type control mice (Fig. 4a, upper panels, and b, upper panel). However, there were more c-Fos^+^ neurons in the basolateral amygdala (BLA) of Math2-Cre;*Hes1* cKO mice than in that of wild-type control mice (Fig. 4a, lower panels, and b, lower panel). We confirmed that there were some Hes1^+^ cells in the BLA of wild-type control mice but approximately 60% less in that of the cKO mice (Fig. 4c). These results suggest that Hes1 may play an important role in the activity of mature neurons in the amygdala.

### Increased sociability and perseveration in inhibitory neuron-specific *Hes1* cKO mice

We next examined the role of Hes1 in inhibitory neurons in the adult brain. To this end, we crossed mice of the *Hes1*-floxed line with those of the Gad2-Cre driver line, in which Cre is expressed specifically in inhibitory post-mitotic neurons^31^. The resulting cKO (Gad2-Cre;*Hes1* cKO) mice (10 weeks old) were subjected to a series of behavioral analyses. These cKO mice were normal in general health, working memory, depression-like behavior, sensorimotor gating, fear, and anxiety (Supplementary Figs S5 and S6), but differed from wild-type control mice in the following tests.

**Figure 5:**
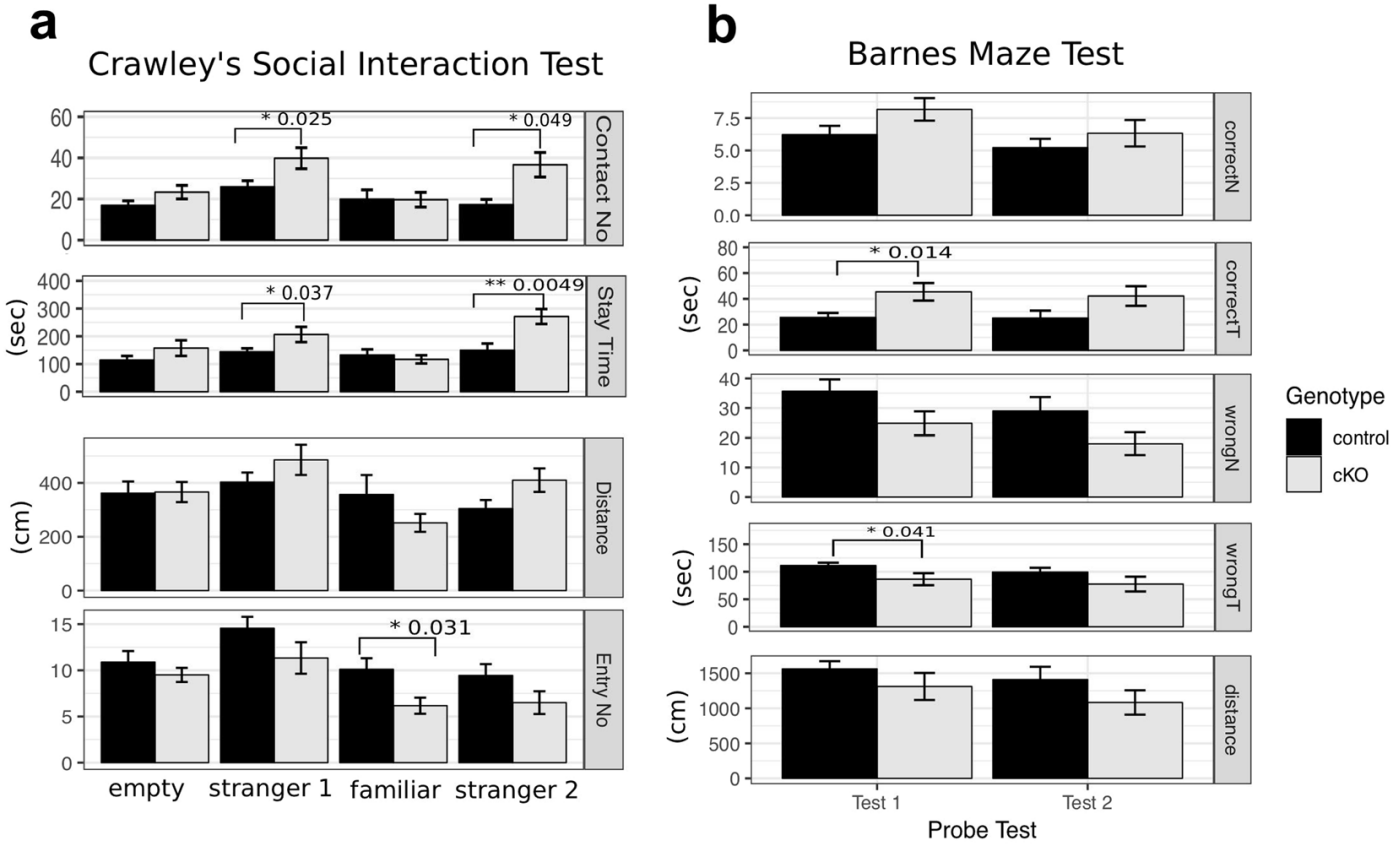
Inhibitory neuron-specific inactivation of Hes1 leads to enhanced sociability and perseveration. (**a**) Crawley’s social interaction test. The number of contacts (Contact No), time spent in each compartment (Stay Time), distance traveled in each compartment (Distance), and number of entries into each compartment (Entry No) were recorded for wild-type control (n = 9) and Gad2-Cre;*Hes1* cKO (n = 6) mice. Data are presented as mean ± SEM; *p < 0.05, **p < 0.01; two tailed Student’s *t*-test. (**b**) Barnes maze test. Probe tests were done on day 1 (Test 1) and day 8 (Test 2) after the training sessions. The number of visits to the correct hole (correctN) and wrong holes (wrongN), time spent in the correct hole (correctT) and wrong holes (wrongT), and total distance traveled (distance) were measured for wild-type control (black, n = 9) and Gad2-Cre;*Hes1* cKO (gray, n = 6) mice. Data are presented as mean ± SEM; *p < 0.05; two tailed Student’s *t*-test.

In Crawley’s social interaction test, a subject mouse was placed for habituation in the middle of a three-chambered apparatus, and then a stranger mouse was placed in the left chamber (see Methods). After a while, another stranger mouse was placed in the right chamber. We found that Gad2-Cre;*Hes1* cKO mice contacted both stranger mice more frequently and spent more time with these stranger mice than wild-type control mice (Fig. 5a), suggesting that the mutant mice showed enhanced social interactions with novel mice.

In the Barnes maze test, spatial memory was measured using a round table with 12 holes, one of which was an escape hole (see Methods). Mice went through training sessions to memorize the position of the escape hole. On day 1 (Test 1) and day 8 (Test 2) after the training sessions, probe tests were performed using the same table but without an escape hole. In Test 1, Gad2-Cre;*Hes1* cKO mice spent a longer time near the escape hole than wild-type control mice, but not in Test 2 (Fig. 5b), suggesting that the mutant mice showed perseveration on day 1.

Together, these results indicated that inactivation of *Hes1* in inhibitory neurons led to increased sociability and perseveration. Thus, Hes1 is functionally important for normal behaviors not only in excitatory neurons but also in inhibitory neurons in the adult brain.

## Discussion

Hes1 is a well-known effector of Notch signaling that regulates the maintenance and proliferation of neural stem/progenitor cells in the developing nervous system. In this study, we first showed that Hes1 is also expressed by both excitatory and inhibitory mature neurons in the adult mouse brain, and that Hes1 expression is induced by neuronal stimulation *in vivo*. Furthermore, inactivation of *Hes1* in excitatory or inhibitory neurons in the mouse brain led to abnormal behaviors, indicating that Hes1 is functionally important in both excitatory and inhibitory neurons *in vivo*. However, it remains to be determined whether Hes1 expression is controlled by Notch signaling in these neurons. Hes1 expression might be regulated by other signaling pathways. For example, cAMP and Ca^2+^ signaling molecules activate CREB transcription factor, which plays an important role in neuronal plasticity and protection^32^. CREB induces Hes1 expression in neural stem/progenitor cells^33^, which raises the possibility that cAMP and Ca^2+^ signaling can also regulate Hes1 expression in neurons, and therefore Hes1 expression might be regulated by various signaling molecules in neurons.

Inactivation of *Hes1* in excitatory neurons of adult mice led to abnormal fear and anxiety behaviors concomitantly with higher neuronal excitability in the amygdala. It was previously reported that Hes1 overexpression in cultured neurons increased GABAergic input^14^; therefore, inactivation of *Hes1* may decrease GABAergic input and increase neuronal excitability in the amygdala. In addition, because Hes1 represses GluR1 expression^14^, inactivation of *Hes1* may increase GluR1-mediated calcium influx and thereby enhance the excitability of neurons. Further study is required to elucidate the mechanism of how the excitability changes in the amygdala in the absence of Hes1 in excitatory neurons, and whether the altered excitability observed in the amygdala is directly or indirectly involved in the behavioral phenotype. The increased anxiety level observed in the open field test was not captured in other analyses, such as the light-dark transition test (Supplementary Fig. S3a,b) and elevated-plus maze test (Supplementary Fig. S4e). Thus, the effect of Hes1 on anxiety is relatively mild, if any, and increased anxiety might not be caused directly by *Hes1* inactivation but rather may be secondary to strongly retained fear memory.

Inactivation of *Hes1* in excitatory neurons led to abnormal retention of fear memory, which is similar to the symptom of posttraumatic stress disorder (PTSD). It is known that even when exposed to the same traumatic stress, some people develop symptoms of PTSD while others do not^34^. This difference may result from different individual characteristics, such as sex, genetics, prior trauma, or age, and Hes1 cKO mice possibly have a predisposition for PTSD. PTSD is a chronic and debilitating disorder, whose pathology is not yet fully understood^35^. A high comorbidity of PTSD with other diseases is making it difficult to accurately understand its pathogenesis. This fundamental lack of understanding of PTSD makes it harder to select or develop effective drugs for this disease. Empirical pharmacological therapies include selective serotonin reuptake inhibitors, which are partially effective^36^. So far, plausible explanations of PTSD pathogenesis consist of monoamine-based and glutamate-based models^35^. Our mice exhibited increased fear memory after we tampered with their excitatory neurons, which may agree with the latter model. Further analysis of Hes1 function in excitatory neurons might shed light on the pathophysiology of PTSD and lead to innovative treatment of the disease. The mouse line we created mainly exhibited enhanced fear memory, which we believe may be useful in elucidating the core pathology of the disease.

We also found that inactivating *Hes1* in inhibitory neurons leads to enhanced sociability and perseveration behavior. The social interaction test and the second probe test of the Barnes maze test also demonstrated a similar tendency, although the difference was not significant. In addition, the cKO mice demonstrated slightly higher stereotypic activity during the first five minutes (p = 0.054) of the open field test (Supplementary Fig. S5e), suggesting that these mice may be hyperattentive. One plausible explanation for these phenotypes is that the hyperattentive state urges the mice to explore stranger mice more carefully, as observed in Crawley’s social interaction test, and to perseverate on their memory, as observed in Barnes maze test. Together, our results indicated that Hes1 expressed in both excitatory and inhibitory neurons plays an important role in the adult brain functions and suggested that two types of *Hes1* cKO mice described here might be useful for analyzing various neurological disorders.

## Methods

### Animals

All animals were handled in accordance with the Kyoto University Guide for the Care and Use of Laboratory Animals. The experimental protocols were approved by the Experimental Animal Committee of Institute for Frontier Life and Medical Sciences, Kyoto University. Mice were housed in a temperature-controlled environment under a 12 h light/dark cycle, with food and water provided *ad libtum*. Generation of *Hes1* floxed (Hes1f) mice and Hes1-Venus mice were described previously^17,18^. Math2-Cre and Gad2-Cre mice^19,31^ were obtained from Dr. Klaus-Armin Nave and Jackson Laboratory (JAX stock #28867), respectively. Excitatory neuron-specific *Hes1* cKO mice were obtained by crossing Hes1f/Hes1f mice with Hes1f/Hes1f; Math2-Cre mice. Inhibitory neuron-specific *Hes1* cKO mice were obtained by crossing Hes1f/Hes1f mice with Hes1f/Hes1f; Gad2-Cre mice.

### Genotyping protocol

For genotyping polymerase chain reaction (PCR), tissues lysed with proteinase K were put into reaction with Emerald Amp master mix (Takara Bio, Japan) using specific primer sets for each genotype. Primer sets used for each genotype were as follows:

*Hes1*-floxed (224 bp for WT, 272 bp for floxed mice)

F: 5′-CAGCCAGTGTCAACACGACACCGGACAAAC-3′

R; 5′-TGCCCTTCGCCTCTTCTCCATGATA-3′

Math2-Cre (525 bp)

F: 5′-GAGTCCTGGAATCAGTCTTTTTC-3′

R: 5′-CCGCATAACCAGTGAAACAG-3′

Gad2-Cre (176 bp)

F: 5′-CACTGCATTCTAGTTGTGGTTTG-3′

R: 5′-AACAGTTTGATGAGTGAGGTGA-3′

Venus-Hes1 (336 bp)

F: 5′-GTAAACGGCCACAAGTTCAGCGTGTC-3′

R: 5′-GTCCTCCTTGAAGTCGATGCCCTTCAG-3′

### Light stimulation and electroconvulsive stimulation

For visual stimulation experiment, each mouse was first kept in a dark cage for 3 consecutive days and then placed in a bright cage with stripe patterns on the wall for 2 hours, after which mice were sacrificed. Control group was kept in dark cages for 3 days and then sacrificed with minimum light exposure.

For electroconvulsive stimulation experiment, each mouse was administered with 0.1 ms, 15 mA square-wave pulse at 100 Hz for 1 second through electrodes placed on both ears using *in vivo* electroporator (CUY21EDIT, BEX Co., Ltd). Mice that showed tonic-clonic convulsion were considered successful and sacrificed 2 hours after stimulation.

### Tissue preparation and immunohistochemistry

Mice were treated intraperitoneally with pentobarbital, and perfused transcardially with 30 ml of PBS and 30 ml of 4% paraformaldehyde (PFA)/PBS (pH 7.2). Brains were post fixed in the 4% PFA/PBS overnight at 4 °C and then cryoprotected for 5 hr in 15% sucrose/TBS and 48 h in 30% sucrose/TBS. Brains were embedded in OCT compound (Sakura Finetek), and frozen at −80 °C. Free-floating brain sections were made at 40 µm thickness with freezing microtome (Leica CM 1950). Sections were incubated in TBS containing 3% H_2_O_2_ for 15 min, 0.5% Triton X-100/TBS for 30 min, 3% normal donkey serum (NDS) and 0.1% Triton X-100/TBS for 2 h at room temperature, then with primary antibodies, rabbit anti-GFP (1:1000, Invitrogen A11122), chicken anti-GFP (1:1000, Abcam ab13970), mouse anti-NeuN (1:200, Merck Millipore MAB377), rabbit anti-Hes1 (1:1000, ref.^37^), rabbit anti-Arc (1:500, Synaptic Systems 156003), and rabbit anti-c-Fos (1:1000, Synaptic Systems 226003) diluted in 0.1% Triton X-100/TBS containing 3% NGS for 3 days at 4 °C. After incubation with secondary antibodies (HRP-donkey anti-rabbit IgG (1:500; GE Healthcare), Alexa 488 or Alexa 594 (1:400, invitrogen) for 2 h at room temperature, sections were reacted with TSA Fluorescein staining solution (Perkin Elmer) for 15 min at room temperature, mounted with Fluormount-G (Southern Biotech) and photographed with laser-scanning confocal microscopy (LSM510, Zeiss).

### Behavioral analysis

Behavioral analyses were performed, as previously described^38^. Mice were genotyped at the time of weaning and group-housed with four mice per cage, two control and two cKO littermates. All the behavioral analysis was performed between 9:00 a.m. and 5:00 p.m.

### General health and neurological screening

Motor function was assessed with wire hang test and grip strength test. In a wire hang test, each mouse was placed on a cage top, which was then inverted to make the mouse hang on to the cage top. Latency of each mouse to fall was measured. In a grip strength test, strength was measured by pulling the tail of each mouse while its front paw grabbing the measurement apparatus.

### Barnes maze

Spatial memory was measured using Barnes circular maze (1.0 m in diameter, 12 holes, O’Hara & Co., Ltd., Tokyo, Japan). Following a habituation phase, each mouse went through 15 training sessions, each lasting for 5 minutes, to memorize the position of one escape hole, which was consistent for a given mouse, but was randomized across mice. After finishing training sessions, 2 probe tests were performed with 7 days interval, each test lasting for 3 minutes. During a training session, the total distance, number of visits around correct and wrong holes, and latency to enter correct hole were recorded. During a probe test, the total distance, number of visits and time spent around correct and wrong holes were recorded.

### Social interaction test

To assess social behavior, a pair of mice was placed in the same cage for 10 minutes. Pairs were chosen with following criteria: same genotype, housed in different cages, and less than 10% difference of body weight. The total duration of contacts, total duration of active contacts, mean duration of contacts, number of total contacts, and total distance traveled were recorded. Active contact was defined as a contact, which occurred after moving speed faster than 10.0 cm/sec.

### Crawley’s social interaction test

Social cognition was assessed using a three-chambered apparatus (TimeCSI for Three-chambered social interaction system O’Hara & Co., Ltd., Tokyo, Japan). First, a subject mouse was placed in the center chamber with the other chambers empty for habituation, and then a stranger mouse was placed in the left chamber to assess general sociability. After that, another yet stranger mouse was placed in the right chamber to assess social novelty recognition. The number of contacts, stay time, distance traveled, and number of entries were recorded for each session.

### Elevated plus maze

Anxiety level was assessed using elevated plus maze, which has one closed arm and one open arm. Each mouse was first placed at the center of the maze and behavior was monitored for 10 minutes. Time spent in each arm, total distance traveled, and number of entries in each arm were recorded.

### Light-dark transition test

Light-dark transition test were performed using an apparatus, which consists of a bright chamber (390 lux) and a dark chamber (2 lux), and mice were allowed to move freely between the two chambers. Time spent in each chamber, total distance traveled, and latency to enter bright chamber were recorded.

### Prepulse inhibition

Sensorimotor gating function was measured by prepulse inhibition test. A 120 dB white noise was used to evoke startle response, and the inhibition of response by preceding prepulse (70 dB, 75 dB, 80 dB, 85 dB) was measured. In addition, a startle response to a single noise (90 dB, 100 dB, 110 dB, 120 dB) was measured to assess auditory and motor function as acoustic startle response (ASR).

### Porsolt forced swim test

Depression-like behavior was assessed using cylindrical aquarium. Each mouse was placed in the aquarium to swim freely and the behavior was monitored for 10 minutes. The experiment was done once a day for two consecutive days. Immobile time and distance traveled were measured.

### Rotarod test

Locomotor activity was assessed using apparatus with a rotating rod (UGO BASILE, VA, Italy). Each mouse was place on top of rod, which rotates at gradually accelerating rate. Latency of the mouse to fall was recorded.

### Y-maze test

Working memory was assessed with a Y-shaped maze, each arm placed at 120 degrees angle from adjacent arms. Each mouse was first placed at the center of the maze and the following behavior was monitored for 5 minutes. The number of entries into arms, alternation rate, and total distance traveled were recorded.

### Tail suspension test

In a tail suspension test, each mouse was hanged by its tail for 10 minutes, and the behavior is monitored. Immobile time was recorded.

### Open field test

Locomotor activity was measured using an open-field test. Each mouse was placed in the corner of the open-field apparatus (40 × 40 × 30 cm; Accusan Instruments, Columbus, OH). The test chamber was illuminated at 100 lux. The total distance traveled, vertical activity (rearing measured by counting the number of photo beam interruptions), time spent in the center area, and beam-break counts for stereotypic behaviors were recorded. The center area was defined as 1 cm away from the walls. Data were collected for 120 minutes.

### Fear conditioning test

Fear memory was assessed with a fear conditioning test. Each mouse was conditioned to associate an auditory cue (55 dB white noise for 30 seconds) with a foot shock (0.35 mA shock for 2 seconds), by administering 3 pairs of an auditory cue and a foot shock. Recall tests were done the next day and certain days after conditioning. In a contextual recall test, each mouse was placed in the same chamber as conditioning but without foot shocks, and behavior was monitored for 5 minutes. In a cued recall test, each mouse was placed in the different chamber as conditioning, and after 3 minutes of observation, the auditory cue was presented for another 3 minutes. Freezing time was recorded in each session.

### Statistical analysis

Statistical analyses were performed using the computing environment R (R Development Core Team). Statistical differences between two groups were analyzed using two-tailed Student’s t-test, and the differences between the two groups at different time points were analyzed using two-way repeated measures ANOVA. P values less than 0.05 were considered to be significant.

## Supplementary information

Supplementary Information
