# Supplementary material for: Hes1 expression in mature neurons in the adult mouse brain is required for normal behaviors
Source: Sci Rep. 2019 Jun 3;9:8251. doi: 10.1038/s41598-019-44698-y (PMC6546782; doi:10.1038/s41598-019-44698-y)
Supplement: Supplementary file 1 — Supplementary Information [file 41598_2019_44698_MOESM1_ESM.pdf]

Hes1 expression in mature neurons in the adult mouse brain is required for normal behaviors

Tadanobu Matsuzaki, Toru Yoshihara, Toshiyuki Ohtsuka, and Ryoichiro Kageyama

Supplementary Information

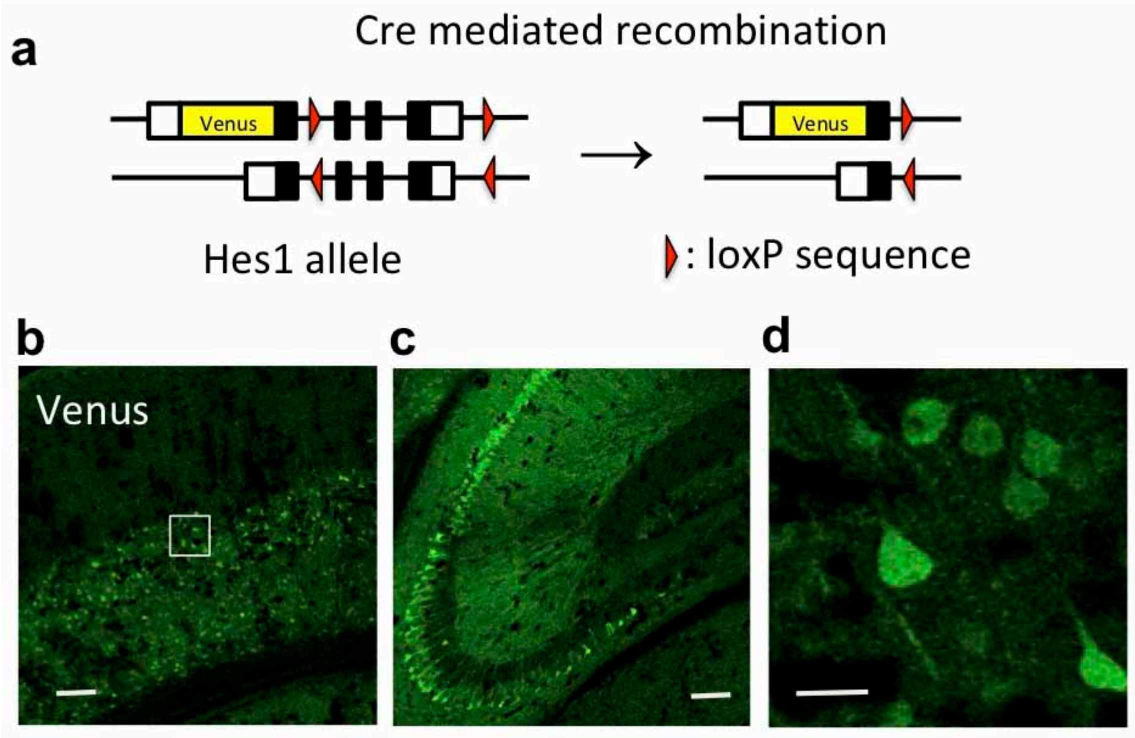

Supplementary Figure S1. Hes1 expression in pyramidal neurons in the adult mouse cortex. **a.** Experimental strategy. In *Venus-Hes1* floxed;*Hes1* floxed;Math2-Cre mice, residual Venus expression occurred under the control of *Hes1* promoter in Cre-positive cells (Math2-expressing excitatory neurons). Because there was no Hes1 protein in such Cre-positive cells, Venus protein should be overexpressed without negative feedback. **b-d.** Immunohistochemical staining for Venus protein. Venus expression clearly occurred in the cortex (**b**) and the hippocampus (**c**) of *Venus-Hes1* floxed;*Hes1* floxed;Math2-Cre mice. Hes1 promoter-induced Venus expression in pyramidal neurons in the cerebral cortex of *Venus-Hes1* floxed;*Hes1* floxed;Math2-Cre mouse (**d**). Scale bars: 100 µm in (**b** and **c**), 20 µm in (**d**).

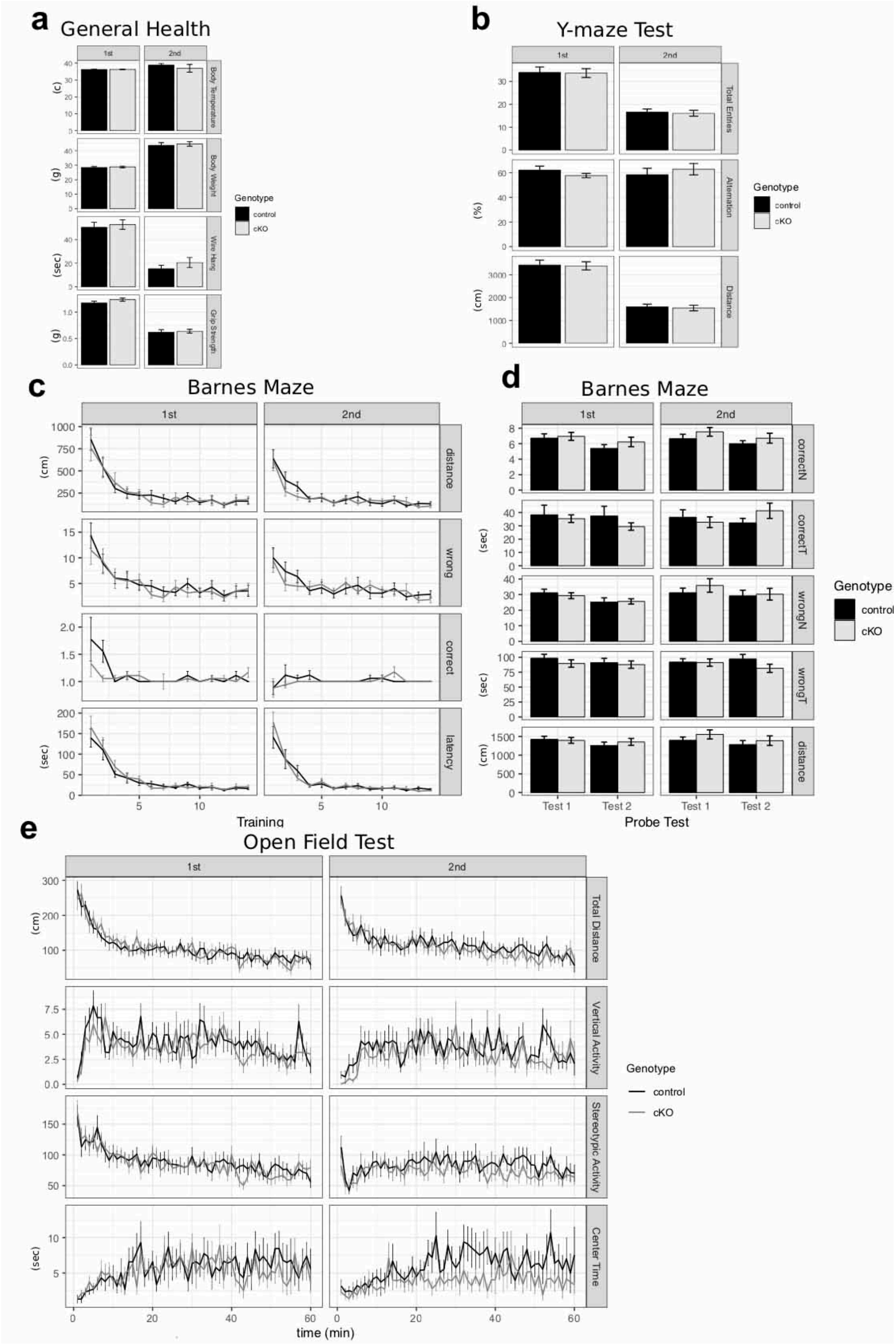

Supplementary Figure S2. Results of behavioral analyses for control (n = 17) and

Math2-Cre;*Hes1* cKO (n = 17) mice. Measurements were conducted in young (10-week-old, left column, indicated as 1st) and old (1-year-old, right column, indicated as 2nd) mice. Data are presented as mean ± SEM. **a.** General health and neurological screening. Body temperature, body weight, latency to fall in the wire hang test, and grip strength were recorded. **b.** Y-maze test. Total entries into arms, percentage of alternations, and total distance traveled were recorded. **c,d.** Barnes maze test. Distance traveled, number of visits to wrong and correct holes, and latency to visit the correct hole were recorded during training sessions. Probe tests were conducted on day 1 (Test 1) and day 8 (Test 2) after the training sessions. The number of visits to the correct (correctN) and wrong (wrongN) holes, time spent in the correct (correctT) and wrong (wrongT) holes, and total distance traveled were measured. **e.** Open field test. Total distance, vertical activity, stereotypic activity, and time in the center were recorded.

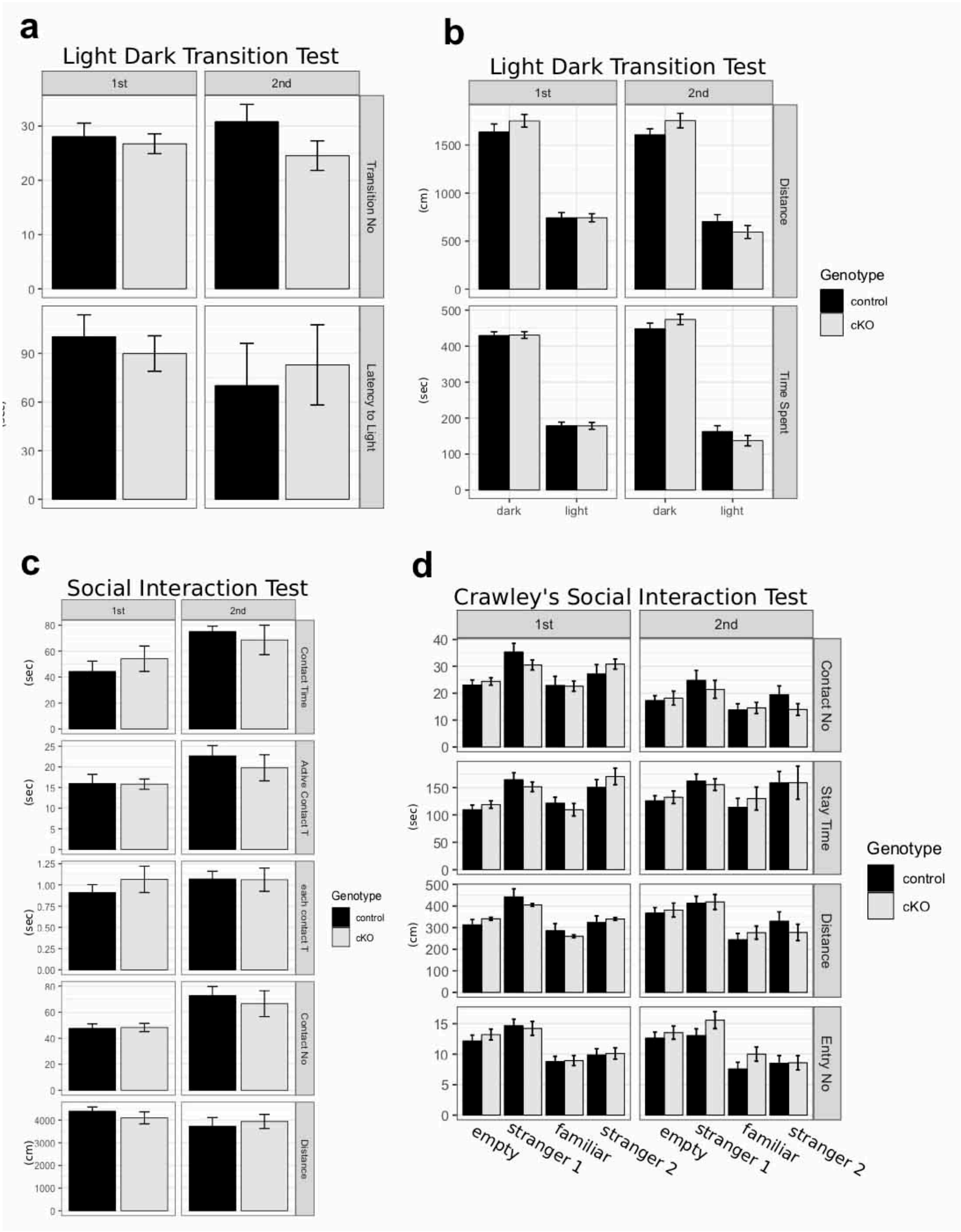

Supplementary Figure S3. Results of behavioral analyses for control (n = 17) and Math2-Cre;*Hes1* cKO (n = 17) mice. Measurements were conducted in young (10-week-old, left column, indicated as 1st) and old (1-year-old, right column, indicated as 2nd) mice. Data are presented as mean ± SEM. **a,b.** Light-dark transition test. The number of transitions between compartments, latency to visit the light compartment, distance traveled in each compartment, and time spent in each compartment were

recorded. **c.** Social interaction test. Total duration of contact, total duration of active contact, duration of each contact, number of contacts, and total distance traveled were recorded. **d.** Crawley's social interaction test. The number of contacts, time spent in each compartment, distance traveled in each compartment, and number of entries into each compartment were recorded.

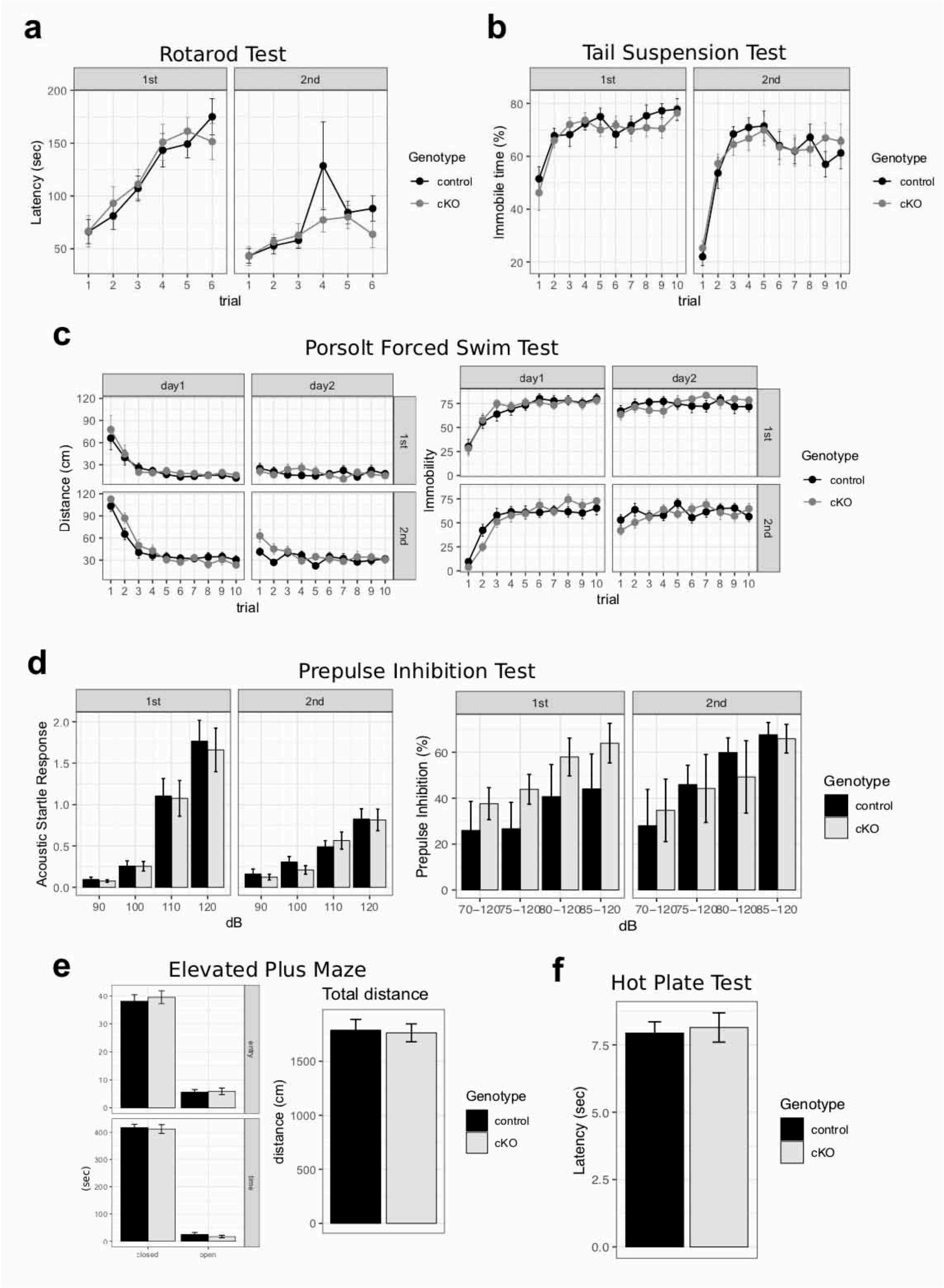

Supplementary Figure S4. Results of behavioral analyses for control (n = 17) and Math2-Cre;*Hes1* cKO (n = 17) mice. Measurements were conducted in young (10-week-old, left column, indicated as 1st) and old (1-year-old, right column, indicated

as 2nd) mice unless otherwise indicated. Data are presented as mean ± SEM. **a.** Rotarod test. Latency to fall was recorded. **b.** Tail suspension test. The percentage of immobility was recorded. **c.** Porsolt forced swim test. Total distance traveled and the percentage of immobility were recorded. **d.** Prepulse inhibition test. Acoustic startle response, and percentage of startle responses inhibited by the prepulse were recorded. **e.** Elevated plus maze. Test was conducted only in young mice. The number of entries into each compartment, time spent in each compartment, and total distance traveled were recorded. **f.** Hot plate test. Test was conducted only in young mice. Latency to fist hind-paw response was recorded.

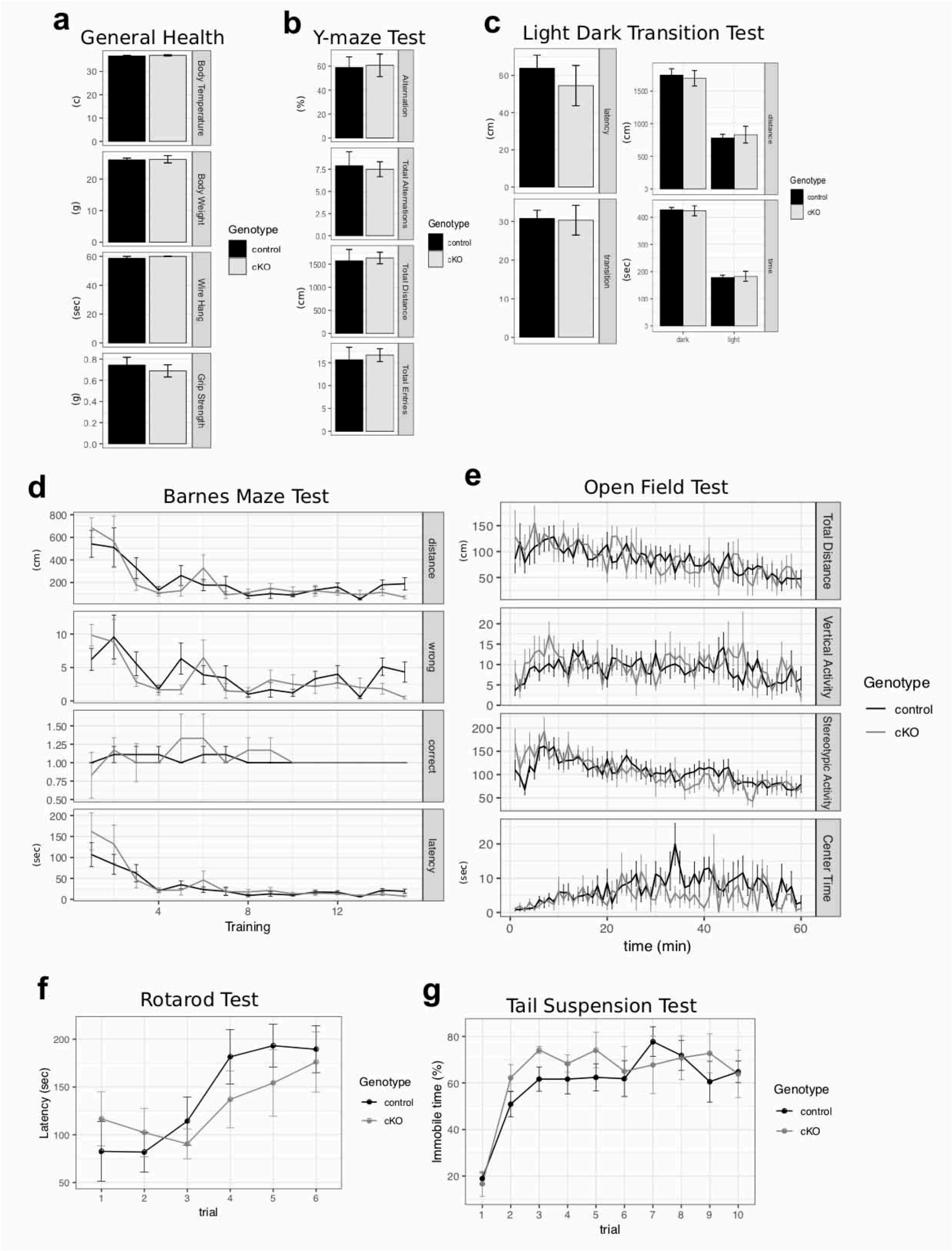

Supplementary Figure S5. Results of behavioral analyses for control (n = 9) and Gad2-Cre;*Hes1* cKO (n = 6) mice. Measurements were conducted in young (10-week-old) mice. Data are presented as mean ± SEM. **a.** General health and neurological screening. Body temperature, body weight, latency to fall in the wire hang test, and grip strength were recorded. **b.** Y-maze test. Total entries into arms, percentage of alternation, and total distance traveled were recorded. **c.** Light-dark transition test.

The number of transitions between compartments, latency to visit the light compartment, distance traveled in each compartment, and time spent in each compartment were recorded. **d.** Training session of Barnes maze test. The number of visits to wrong and correct holes, distance traveled, and latency to visit the correct hole were recorded during training sessions. **e.** Open field test. Total distance, vertical activity, stereotypic activity, and time spent in the center were recorded. **f.** Rotarod test. Latency to fall was recorded. **g.** Tail suspension test. The percentage of immobility was recorded.

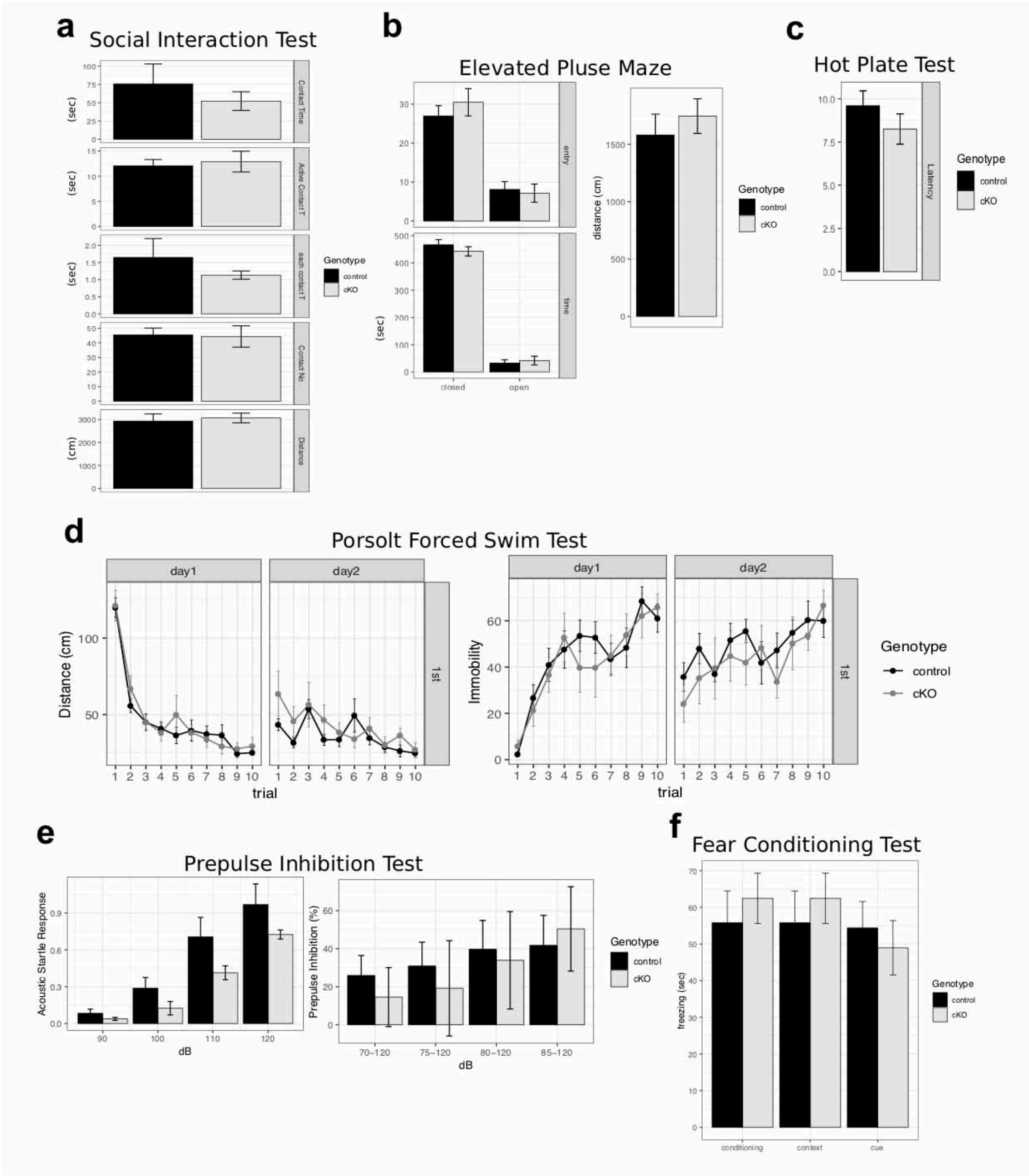

Supplementary Figure S6. Results of behavioral analyses for control (n = 9) and Gad2-Cre;*Hes1* cKO (n = 6) mice. Measurements were conducted in young (10-week-old) mice. Data are presented as mean ± SEM. **a.** Social interaction test. The total duration of contact, total duration of active contact, duration of each contact, number of contacts, and total distance traveled were recorded. **b.** Elevated plus maze. The number of entries into each compartment, time spent in each compartment, and total distance traveled were recorded. **c.** Hot plate test. Latency to fist hind-paw response was recorded. **d.** Porsolt forced swim test. The total distance traveled and the percentage of immobility were recorded. **e.** Prepulse inhibition test. Acoustic startle response, and percentage of startle responses inhibited by the prepulse were recorded. **f.**

Fear conditioning test. Times spent in “frozen” position during fear conditioning, context recall test, and cued recall test were measured.
